# Neurodivergence and the Rabbit Hole of Extremism: Uncovering Lived Experience

**DOI:** 10.1089/aut.2023.0192

**Published:** 2024-08-09

**Authors:** Sachindri Wijekoon, John Robison, Christie Welch, Alexander Westphal, Rachel Loftin, Barbara Perry, Victoria Rombos, Christian Picciolini, Catherine Bosyj, Lili Senman, Patrick Jachyra, Simon Baron-Cohen, Melanie Penner

**Affiliations:** 1 Western University, London, Canada.; 2 William & Mary Williamsburg, Williamsburg, Virginia, USA.; 3 University of Toronto, Toronto, Canada.; 4 Division of Law and Psychiatry, Yale School of Medicine, New Haven, Connecticut, USA.; 5 Department of Psychiatry and Behavioral Sciences, Feinberg School of Medicine, Northwestern University, Chicago, Illinois, USA.; 6 Ontario Tech University, Oshawa, Canada.; 7 Bloorview Research Institute, Toronto, Canada.; 8 Free Radicals Project, Petoskey, Michigan, USA.; 9 Department of Sport & Exercise Sciences, Durham University, Durham, United Kingdom.; 10 Department of Psychiatry, Autism Research Centre, University of Cambridge, Cambridge, United Kingdom.

**Keywords:** autism, autism spectrum disorder, extremism, hate group, radicalization, vulnerability

## Abstract

**Background:**

There have been sporadic and disturbing media accounts of autistic people engaging with extreme ideologies, with comparatively little systematic exploration of this suggested association. Existing research has failed to consider the contextual factors that could influence these rare occurrences of engagement with extreme ideologies. This study explores how autistic individuals involved in extreme ideologies describe personal and contextual factors affecting their participation.

**Methods:**

Twelve individuals from Canada and the United States who were either diagnosed or self-identified as autistic and have engaged with extreme ideologies participated in semistructured interviews. The research approach and analysis of the data were informed by interpretative phenomenological analysis. Our interdisciplinary team met regularly to collectively examine initial assumptions and interpretations, while maintaining a central focus on the perspectives of the participants.

**Results:**

We identified the following three key themes: (1) early wounds, (2) missed formative opportunities, and (3) finding a fit for neurodivergence. Traumatic experiences, disenfranchisement, learned hatred from an insular upbringing, and systemic failings in health and social service systems contributed to participants’ decisions to engage with extreme ideologies. Hate groups, in turn, filled the voids by providing acceptance, purpose, structure, sense of community, and by accommodating participants’ neurodivergent needs.

**Conclusion:**

Autism alone did not explain participants’ engagement with extreme ideologies. Trauma and disenfranchisement related to being neurodivergent were common factors that made hate groups more appealing. Proactive interventions to prevent engagement in extreme ideologies must champion inclusive environments that recognize autistic individuals’ skills and address underlying factors that contribute to their disenfranchisement.

**Community Brief:**

**What was the purpose of this study?**

The media has reported on high-profile cases of autistic people with extreme beliefs who acted in violent ways. There is a lack of research on this topic and researchers have not directly spoken with autistic people who have been involved with these extreme beliefs. Our goal was to understand why some autistic people engaged with hateful beliefs, asking them about both autism and their life circumstances.

**What did the researchers do?**

We interviewed 12 people who identified as autistic to understand why they became involved in extreme beliefs. We conducted the interviews by phone or using Zoom Health. We read the interview text, identified important statements, and then identified the ideas linking these statements.

**What were the results of the study?**

Most of the people in our study were young to middle-aged men with White/European background from Canada and the United States. Only a few had a formal autism diagnosis.

Participants faced many challenges, including being neglected by parents, experiencing trauma, and not feeling like they belong. Many of them were not given opportunities to freely express themselves or have positive interactions with people from different backgrounds. Everyone lacked the opportunity to build a positive view of themselves and the world around them.

Participants described some autistic and neurodivergent traits, such as having a focused interest in one topic, having difficulty understanding and connecting with others, preferring clear rules and a set routine, and having difficulty controlling emotions, were not accepted elsewhere but were accepted in groups with extreme beliefs.

**What do these findings add to what was known?**

Hate groups provided autistic people a supportive environment where their strengths were highlighted, their individuality was celebrated, and their challenges were accommodated. This level of support contrasted with what autistic people had previously experienced in society.

**What are potential weaknesses?**

One limitation of our study was that we included people who identify as autistic, but we did not verify their diagnosis through formal testing. Our sample is a small group of autistic people who had engaged with hateful beliefs; our findings do not apply to all autistic people and should not be interpreted that all autistic people are more likely to have extreme or hateful beliefs.

**How will these findings help autistic adults now or in the future?**

Engagement with extreme groups or beliefs is only one possible negative outcome from the lack of inclusion and acceptance of autistic people, but is an important one. We should create supportive environments that welcome and appreciate autistic peoples’ skills and interests to allow them to feel valued and connected to their community. Families, teachers, and professionals should prioritize accurate and timely diagnosis and provide supports that are tailored to their needs. These steps can help autistic people build meaningful relationships and prevent them from turning to extremist groups to meet their needs.

## Introduction

The pervasiveness of extremism in North America is marked by numerous hate groups.^1^ Extremism, defined as beliefs that deviate from mainstream views and often advocate for radical changes in government, religion, or society,^2^ can result in threatening or destructive behavior toward those who oppose the extremist’s worldview.^3,4^ Various forms of extremism exist—left-wing extremism, which advocates for a socialist agenda; right-wing extremism, which displays antiglobalism, racial or ethnic supremacy, or nationalism; and religious extremism, which involves violence in support of a particular faith-based belief system.^5^

Sensationalized media reports have suggested without evidence that there is a link between extremism and neurodivergent conditions, particularly autism. Autism is complex and heterogeneous; autistic individuals may have specific social needs and interaction styles that are not understood within a neurotypical society. They may also experience the sensory world in different ways, have strong interests, and engage in repeated behaviors. Many aspects of autism represent strengths in the right context.^6^ Because of this, extremist groups have sought to recruit autistic individuals to utilize their strengths,^7^ often with a stereotypical view of their technological prowess and affinity for repetitive tasks. Work from our group exploring use of the term “weaponized autism” on an alt-right social media platform showed that autistic people were described as “ripe” for exploitation. This was less due to the core features of autism and instead related to the disenfranchisement of autistic people, who are cynically described as “NEET” (not in education, employment, or training).^8^

Robust data on rates of engagement with extreme ideologies among autistic or otherwise neurodivergent people are not available. At the population level, neurodivergent individuals are no more likely to commit acts of violence compared with the general population and are often victims of violence.^9^ One UK-based report showed that a notable proportion of individuals in counter-radicalization programs exhibit autistic traits.^10^ However, some have warned that this overrepresentation may instead suggest that certain features associated with autism, such as intense interests and drive to collect information, might be mistakenly interpreted as signs of radicalization.^11^ A recent literature review^12^ on autism and extremism reported low quality of evidence, stating that existing research cannot conclude that autistic individuals are more susceptible to involvement in terrorism than the general population. Some studies^9,13^ offer interpretations attributing engagement to facets of autism, overlooking contextual factors. Existing research in this area has primarily included case studies, and none has consulted autistic individuals directly involved in extreme ideologies. Despite the absence of definitive evidence between autism and susceptibility to extremism, high-profile mass violence events involving perpetrators diagnosed or described as autistic have drawn media attention, thus stigmatizing the broader autistic population. Drawing direct conclusions about the association between neurodivergence and susceptibility to extremism is irresponsible^14^; as such, further contextual exploration is essential to understand these rare cases of autistic people who engage with extreme ideologies. This study investigates how individuals who identify as both autistic and involved in extreme ideologies describe their experiences of autism, their social networks, and the broader societal context.

## Methods

### Research paradigm and positionality

We used an interpretive lens to delve into multiple realities and subjective meanings, and drew on a relativistic epistemology, acknowledging that reality and meaning are not fixed but rather shaped by individual and cultural perspectives.^15^

We conducted this research from multiple research and clinical perspectives (e.g., developmental pediatrics, psychiatry, occupational therapy, psychology), guided by an autistic advocate and a former extremist. Our team unequivocally opposes the ideas presented in the extreme groups in which our participants engaged. We believe that autism itself does not lead to hate or violence. We have tried to balance fidelity to our participants’ lived experiences while also reflecting on forces that shape the stories they told us. While we discuss the factors that influenced their engagement, we do not believe that participants lacked agency in choosing to participate in extreme ideologies.

The research team engaged in reflexivity to enhance the credibility and trustworthiness of our findings. Monthly peer debriefing with the research team and external checks with two autistic advocates contributed to the interpretive process. S.W. and M.P. met biweekly to discuss the analysis. We use vivid descriptions to present a nuanced and authentic portrayal of the experiences of study participants.

### Design and participants

The study was situated within an interpretative phenomenological analysis (IPA) approach, which guided data generation and analysis.^16^ IPA draws on phenomenology, hermeneutics, and idiographical (i.e., prioritizing understanding the unique characteristics of each individual) underpinning to explore how individuals understand and interpret life experience.^17^ IPA seeks to uncover intrasubjective (individual) and intersubjective (group) meanings generated within the context of their life experiences, examining how individuals make sense of their personal and social world.^16^ It is useful for understanding the varied interpretations and experiences of heterogeneous groups, such as the autistic community.^18^

We recruited 12 individuals who self-identified as autistic or were formally diagnosed, and previously engaged in extreme ideologies. The autistic community recognizes the validity of a self-diagnosis,^19^ in part, because obtaining a formal diagnosis is difficult for adults.^20^ We use the term “engagement with extreme ideologies” to describe activities across all participants and use the term “hate group” for engagement with other members, whether online or in person. We recruited participants via a founder of an organization that helps people leave hate groups, recruitment posts on X (formerly Twitter), and our professional networks.

### Data collection

Senior author, M.P., conducted semistructured interviews via Zoom Health or telephone, ranging from 60 to 180 minutes. The research team provided input in developing the interview guide. In keeping with IPA’s pursuit of insider perspectives, semistructured interviews gave participants a voice^21^ and facilitated rapport-building between researcher and participants. Participants had agency over the interview pace and direction.^18^ Interviews were recorded and transcribed verbatim. We determined the sample size at the point of information redundancy, where no new ideas emerged.

### Data analysis

The first author, S.W., read, reread, and made conceptual and linguistic exploratory notes for each transcript, using NVivo 11.^22^ S.W. then created and categorized “personal experiential themes,” searching for connections across them guided by the principles of IPA. Finally, S.W. named the themes and consolidated them, repeating this process across transcripts to create group experiential themes.^16^ We engaged in reflexive analytic practices by examining our values and preconceptions. Regular team discussions allowed us to challenge initial assumptions and interpretations. Simultaneously, we considered the perspectives of our participants, ensuring their experiences remained central to our research.

### Ethical considerations

All participants provided informed consent before interview scheduling. At the beginning of the interview, we reminded participants of the confidentiality and anonymity of their data, including limitations on confidentiality. We reviewed necessary actions if the participants disclosed plans to harm themselves or someone else. We asked participants to disclose their location so that we could act if such information was disclosed. Location information was destroyed immediately after the interview. Interviews were conducted by an experienced clinician researcher. If participants disclosed distress that did not require immediate action, we provided information about national hotlines for support. We removed any identifiers, such as names, affiliations, and specific organizational details to safeguard identities. Participant quotations were identified using pseudonyms. Team members involved in conducting interviews, transcribing, and analyzing the data held regular debriefing sessions to support their mental health. The study was approved by the Holland Bloorview Research Ethics Board.

## Results

### Participant characteristics

Most participants (*n* = 9) were aged 25–44, identified as male (*n* = 8) or nonbinary (*n* = 3), and as White/European (*n* = 12). Only five (*n* = 5) had a formal autism diagnosis. Co-occurring conditions were common (Table 1).

All of our participants are former far-right extremists. Their involvement varied between active participation (e.g., leading and attending rallies, participating in online forums) and exclusively passive engagement (e.g., listening to neo-Nazi music and researching Nazis online). Participants described distressing experiences, including abuse and sexual assault. Reader discretion is advised.

We identified the following three interconnected themes: (1) early wounds, (2) missed formative opportunities, and (3) finding a fit for neurodivergence.

### Theme 1. Early wounds

Trauma was a pervasive experience in our sample. Participants reported attachment disruptions, abuse, and marginalization within a neurotypical society from childhood. This theme explores how these adverse experiences shaped their worldview and created susceptibility to far-right hate messaging.

#### A traumatic childhood

Participants shared a common thread—an overwhelmingly traumatic childhood. Compound traumas included physical abuse, emotional neglect, sexual exploitation, and exposure to violence and substance abuse.

My adopted father passed away the year after I was born. It was turbulent. I was molested as a kid… there was a lot of substance abuse, like get drunk fast enough to like each other. —Jesse

Attachment disruptions were prevalent, including parental neglect or abuse, separation (including death) from guardians, and insufficient attention from family or professionals, creating feelings of isolation and vulnerability.

My mother had just passed away when I was 14, suddenly passed away. My two brothers were much older than me. So, I was this lonely latchkey kid coming home to an empty house. —Casey

Participants reported disruptions to their attachment systems had far-reaching implications, including mental health issues, substance abuse, and self-harm. Their sense of abandonment caused many to search for belonging outside of their family.

#### A catalytic event

Several participants described how a traumatizing experience altered their view of safety and trust. One participant (Jesse) described how an isolated incident can inspire animosity to an entire group.

A lot of these guys had some really tragic story that happened to involve someone of another race, that it gave them a direction to point their anger. X when he was like 10 years old [was] walking home from school. Some older kids just beat the shit out of him, and actually broke his face so bad that the front portion of his face was a plate that he could remove.[This incident] had a long-lasting effect that shaped his view. —Jesse

**Table 1.** participant demographics

	n
Country	
United States	11
Canada	1
Ethnicity	
White/European^a^	12
Age	
18–24	2
25–34	5
35–44	4
55–64	1
Gender	
Man	8
Nonbinary	3
Other: “assigned female/male at birth”	1
Formal autism diagnosis	
Yes	5
No	7
Marital status	
Single	7
Divorced	3
Married	2
Education	
High school or GED	1
Trade school	1
College courses	2
College degree	3
Associate’s degree	2
Master’s degree	2
Professional degree	1
Employment	
Employed, full time	7
Student	2
Employed, part time	1
Unemployed	1
Prefer not to say	1
Income	
≤40,000	5
40,001–80,000	3
80,001–125,000	3
Prefer not to say	1
Geographical location	
1,000–29,999 people	5
30,000–99,999 people	3
≥100,000 people	4
Co-occurring diagnoses	
Anxiety disorder	6
Attention-deficit/ hyperactivity disorder	6
Depression	4
Personality disorder	3
Acquired brain injury/concussion	2
Other (each *n* = 1): learning disability; obsessive-compulsive disorder; oppositional defiant disorder; genetic disorder, alcoholism, cyclothymia	6

a One participant specified Irish descent and another Scotch/Irish.

Another participant described the interplay between an upbringing characterized by hatred and an incident that reinforced preexisting biases. The traumatic event was seen as watering a seed already planted within the participant’s consciousness.

When I was a sophomore, I was raped by a Hispanic gentleman. He wasn’t a gentleman. He was an asshole. But you know what I mean. I think as a coping strategy, some little seed that I thought I had eradicated started to germinate again.… And then, right after I graduated from school, I got raped again. This time it was by a black guy. And I was like, well shit. Like it was just a layer in my brain that was built upon these sedimentary layers of trauma at the hands of a person of color. So I guess autism kind of paves the road, but there has to be some catalytic event that starts the car and gets you going.—Rene

This participant felt that while being autistic shaped their experiences leading to hate group participation, on its own it did not explain their involvement.

#### Victimized in a neurotypical world

Participants described being marginalized, abused, and exploited in a neurotypical society. They struggled with living in a world tailored to neurotypical norms and preferences: “The only reason that we’re divergent and a problem is because we don’t fit your box, and we know it.” (Robbie)

Participants experienced “an ocean of bullying and condemnation, failure, disappointment, and frustration” (Robbie) early in life due to being autistic. Jesse illustrated this, stating: “Throughout my life there have been a lot of feelings of being unwanted, not good enough, rejection, not feeling like I can find my place, not feeling like I measure up.” They were undermined by messaging from within their innermost social circle, with Parker recounting an instance when his mother said “You are autistic. You shouldn’t be able to drive. You shouldn’t be able to leave the house.”

Participants encountered discrimination by professionals, including educators and health care providers. Parker described an instance when a teacher said autistic people “are gonna get stuck with labor jobs.”

Participants’ encounters with marginalization influenced their self-perception and shaped beliefs about their worth and capabilities.

Kids would bully me all the time as soon as they found out.… I was embarrassed that I had autism. It was like a lot of time, me just internalizing all of that like, “Hey. You’re dumb as hell. You’re never going to be able to do anything,” because everyone around me says the same damn thing.—Parker

The stigmatization of participants fostered grievances and resulted in feelings of being “alone against the world” (Eli). Casey describes being a “lonely kid who had a lot of anger issues with people after the way that they had just made fun of [me] and mistreated [me] all of those years”. Another participant expressed,

People thought I was a school shooter because I didn’t know how to talk to people. They were afraid of me. Everyone just picked on me. They didn’t know I was autistic, but because I acted differently, they figured I was r*****ed. They were just picking on me all the time. I was very isolated. I was angry at the world. —Parker

For some, this anger manifested as indignation or a desire for retribution.

I didn’t have friends until I was 14 years old. You can imagine how difficult that is. I remember that incredible real deep resentment that became total hatred, and the planning, and a really skewed reasoning. I suffered with that kind of anger for about 6 years. I was even violent with my friends.—Robbie

Participants’ experiences fueled anger and longing for acceptance, prompting them to seek out inclusive spaces that valued and empowered them “I just never felt like I belonged in society, and so I would look for the groups that would take the outliers.” (Hunter)

### Theme 2. Missed formative opportunities

This theme discusses how participants missed opportunities to develop a positive, integrated understanding of themselves and the world due to an insular upbringing, absence of a formal autism diagnosis, and being unheard and misunderstood.

#### An insular and hateful upbringing

Many participants grew up in White-nationalist environments and internalized these ideologies.

I grew up in a religious right family that also has neo-Confederate ideology. We would pass by a yard that had the Third National Confederate^1^ battle flag flying in their yard, and my dad would get all pumped up. —Hunter

Bigotry was a pervasive part of participants’ upbringing, with hate symbols accessible in their homes: “I remained in a community where people’s grandparents still had their [Ku Klux Klan] hoods up in their attics” (Hunter). Participants were exposed to bigoted discourse: “My grandfather on my father’s side will not hesitate to say the N word. [It] didn’t matter who was around.” (Avery) Some were introduced to organized racism early on: “I remember being like six when my dad took me to my first Klan rally.” (Rene)

Guardians hid participants from diverse people and perspectives, such as enrolling them in segregated schools (Hunter) and taking legal action against desegregation efforts (Casey). Rene’s family friends rented an entire neighborhood to exclude people of color. Thus, participants largely remained in ideological echo chambers, only encountering diverse individuals, behaviors, and cognitive frameworks later in life.

#### Slipping through the cracks

Participants discussed the under-recognition of neurodivergent features by parents and professionals, resulting in a failure to seek formal diagnoses and professional assistance. Most participants had not been formally diagnosed as autistic, but rather self-identified based on their experiences of feeling *weird* (Hunter) or *eccentric* (Avery), not fitting in with crowds, and being unable to form or maintain social relationships.

Many described having features of autism dismissed by parents and other adults.

[My parents] were like, “we know the autistic kids…they bang their heads against the wall and stuff like that.” They’re like “our kid is not autistic. And if you say it again, we’re gonna flip our lid on you … because autistic people have terrible lives, and they never hold jobs.” And so [parents] just didn’t acknowledge it. —Rene

Participants recounted feeling invalidated when seeking support from adults, who responded with frustration and disciplinary measures.

My dad was always against that stuff you know, like I had been telling him since I was in third grade, that I thought I had [Attention Deficit Disorder]. And he used to say there is no such thing, only kids without discipline, and he’d hit me with his belt. —Hunter

This lack of empathy from others led to prolonged distress, self-doubt, and difficulty forming an identity.

I’d come home crying every day, asking my parents to put me in a different school, and they’d refuse. They’re like ‘you just have to learn how to get along’, but never taught me how to get along. In the fall of my freshman year in high school, things had gotten so bad, and they were in the car on the interstate berating me, and I couldn’t escape. So, I opened up the car door and told them I was gonna jump out onto the interstate and kill myself to get away from them. They stopped me, and then my mom decided they needed to take me to see the psychiatrist.—Hunter

Guardians’ denial of their child’s diagnosis prevented advocacy and communication with schools, leading to misunderstanding and lack of support.

In my early childhood, people knew I had something. I was put in special education in preschool. It wasn’t until the end of second grade when they got me diagnosed with autism spectrum disorder. My mother was meeting with my second-grade teacher … and she would say “well, there might have been mention of possible [pervasive developmental disorder]”. I get the impression that my mother just didn’t want to get me diagnosed back then because she didn’t want to accept what I had. —Alex

Oversights in the assessment and referral practices of clinicians and teachers were discussed. Cameron noted: “They just thought I was weird, but a good teacher would have tried to help me.” Participants cited a lack of proactive measures to access supports, despite their neurodivergence being evident.

Everyone that I was at school with called me autistic. My guidance counselors were always suspecting like ‘you could be autistic’…[But] I didn’t get diagnosed with [Attention-Deficit/Hyperactivity Disorder] until maybe 4 years ago. So, it’s really been a lifelong struggle to figure out what is up with me. —Morgan

Undiagnosed participants searched to understand their behavioral patterns, longing for clarity an earlier diagnosis could have provided, including validation of their struggles and earlier access to self-compassion.

They also recognized how health and social service systems were ill-equipped to provide support and accommodations for neurodivergence. Rene stated, “But I think it would have been worse for me, if my path had been set as being diagnosed as autistic because we just didn’t have the infrastructure.” Others discussed being caught in a cycle of professional handoffs, highlighting the inefficiency of the health care system, such as Hunter, who estimated seeing 16 different psychiatrists.

#### My feelings never mattered

Participants discussed invalidation of their emotions, which caused them to doubt the validity of their experiences and hindered development of healthy coping mechanisms. The lack of validation drove them to seek external affirmation wherever they could find it.

Several participants described not being afforded the space to process traumatic events. Casey discusses the lack of acknowledgment of his emotional needs following the death of his mother: “I remember my father telling me how proud he was that I didn’t shed a tear at my mother’s funeral. That was a good thing.” These forms of invalidation resulted in feeling misunderstood and emotionally isolated.

…it’s really hard to grow up in a world that thinks it understands you, but doesn’t. …for me the root of a lot of my anger and rage was that it felt like my feelings, my thoughts never mattered. What other people wanted to do was always going to supersede the thing that made me comfortable and feel safe. —Jesse

Participants expressed feelings of invalidation due to a lack of understanding from authority figures. Instead of providing support, these figures often responded with frustration, exacerbating participants’ feelings of isolation: “I had trouble keeping up and focusing in class and my parents didn’t understand why. They would spend a lot of time lecturing and yelling at me.” (Alex). Some internalized the perceptions of teachers and parents as being rebellious and assimilated to this role.

This was another big part of how I got involved—my parents were always telling me that I was just rebellious and hateful when really that wasn’t really the case. But then, after so long, I remember consciously saying, well, if they just think that I’m rebellious and hateful, then I’ll just be rebellious and hateful. —Hunter

### Theme 3. Finding a fit for neurodivergence

Participants reported certain characteristics of their autism and neurodivergence, such as hyperfixation; unmet social needs; preference for rules, routines, and structure; and emotional dysregulation influenced their engagement with extreme ideologies. They highlighted that these same personal traits had led to exclusion in the neurotypical world. By contrast, these characteristics were accepted, accommodated, and even celebrated in hate groups. The relative reverence for autism within the hate group empowered participants, offering a stark contrast to the marginalization they experienced in mainstream society.

There was a sense in which my autism was valued. Looking back, I’m now realizing that there was also a sense in which they just wanted the benefits of it, but overall you’re still less than us. It’s a deceitful tactic, but I think they mainly preyed on the fact that I felt marginalized. —Avery

#### An all-consuming interest

Several participants described intense, exclusive interests in topics such as serial killers, World War II, Nazis, personal heritage (e.g., Irish, Nordic), and religion (e.g., Paganism, Christianity), investing hours and cognitive resources to gain mastery: “I have broad interests, but I just hyper focus on these things. So, if White nationalism is the new thing, I’m basically going to be able to quote a book to them.” (Hunter)

Participants reported that their initial interest in benign topics, such as Norse mythology (Avery), expanded to associated topics, leading them to dangerous communities (e.g., White supremacist forums), reportedly without their intent: “I was looking into Norse paganism at the time … This is kind of what led me down the road of getting me to these White supremacists [forums].” (Avery)

Some participants reported detaching from reality as they went down the “rabbit hole” (Rene), into extremist worldviews that aligned with their interests. These interests became the focal point of their lives at the expense of other important things, including social relationships: “It was hard for me to talk about anything else, which made it difficult to socialize with others. I’d read about it constantly whenever I had spare time.” (Avery)

Hyperfixation also facilitated social connections within hate groups. Immersion in their interests made participants confident and knowledgeable when discussing these topics, leading to conversations and camaraderie with like-minded individuals.

Within the group itself, I had a couple of good [friends]. Eighty percent I’d say of the guys who were in the [group] were both intelligent and interested enough to have theoretical, idea-based conversations. —Rene

#### Finding desired connections

Participants expressed discomfort in mainstream social situations, struggling to navigate norms, understanding sarcasm and humor, inferring others’ mental states, and integrating different perspectives. They reported retreating from mainstream social interaction due to anxiety and sensory sensitivity, resulting in loneliness and isolation.

[Social isolation is] one of the biggest things in my life. . . . I’m way more isolated than I want to be, or else I’m trying to do something about that and become overwhelmed. . . . I’m mostly just, well, right here, this is where I sit every day and study in this spot, maybe like eight hours a day. —Hunter

Hate-based propaganda and groups seized upon the struggles of participants who struggled to form meaningful relationships and find acceptance elsewhere.

I would have joined the youth group of the church if they had shown us any interest at all. But they never did. The [hate group] came along and said, “Hey we’ll be your family, and no one will ever make fun of you again, and you will have power.” Well, that’s pretty enticing. —Casey

All participants desired a sense of community and social connection; for those who were recruited, the invitation to join a community was irresistible: “We don’t usually fit in anywhere. So don’t hand us a full invitation because we will just be like “okay. Now I’m a part of something. I’m going to go along with you” (Robbie). Participants sought spaces that satisfied their need for social interaction without requiring adherence to neurotypical standards.

There were a few leaders [in the hate group] that kept everyone else away from me. They were obviously aware that I got a little bit shook talking to strangers. My interactions were with these five or six people for the most part, and that made me feel comfortable because I got to know those people and what to expect from them. They knew it and so they protected me from being around too many people in one-on-one situations. —Casey

Autistic features, which might have been cause for past social exclusion, were suddenly prized attributes:

His second in command had a bad memory and kind of wanted help in that particular area. But also, my ability to retain information, when I go through the curriculums and I would read the [Norse mythology texts].… And I think he got so mad that I wasn’t able to show up at that meeting because I would have been able to explain it to a lot of these newcomers in a way that was very simple to understand. —Avery

Within the hate group, participants developed deep bonds, viewing the community as a “brotherhood” (Eli). This familial language reinforced a shared purpose, identity, and loyalty to one another: “And the thing is, the [hate group] taught us that anyone who isn’t in the [hate group] is an alien, and we owe nothing to aliens, whether they’re blood relatives or not.” (Casey). This indoctrination facilitated the formation of new friendships, providing novel social acceptance for participants, and was reinforced through strong in-group messaging.

Overall, hate group environments provided a sense of unconditional understanding, where other members were perceived to comprehend their struggles and provide empathetic support, reinforcing the allure of membership as a source of genuine connection that had previously been lacking.

#### A predictable environment

Participants had a strong preference for structure and predictability, manifesting in their need for routines, clear expectations, and adherence to rules and regulations. Having clear expectations for behavior enabled navigation of social interactions.

I think I liked that there was a sense of order because even when we were out being violent, we had a code of conduct and rules. So, the entire time I was with them, I didn’t do meth because that group had the viewpoint that drugs were just polluting your perfect Aryan body. —Jesse

The structured environment of hate groups, including their hierarchy and rules, appealed to participants’ need for structure and predictability. Structure was provided through ideological narratives, rites of passage, dress codes, and grooming standards: “I know what I’m supposed to do, where I’m supposed to be, and in a lot of ways sometimes what I’m supposed to believe.” (Jesse). This created stability and control for participants who found comfort in having clear expectations.

The ritual and the regalia, and the hierarchy of white extremism … like there is a uniform. I don’t have to think about it … that really resonated with me. There’s a black nylon bomber jacket, and everyone wears the same patch in the same place on your bomber jacket … everything is the same every single time and that is very soothing. —Rene

Hate groups’ predictability attracted participants who craved certainty in an uncertain world, alleviating their anxiety around social decision-making with the simplicity of predetermined beliefs and behaviors.

#### Experiencing emotional resonance

Participants reported difficulty controlling emotions, leading to disruptive outbursts starting in early childhood. They described receiving messages that these feelings were prohibited and needed to be suppressed.

I was a socially awkward kid who had a lot of anger and a lot of rage but then for most of my life was told that I wasn’t allowed to have it and that to give into it was something I just wasn’t allowed to do. —Jesse

Participants attributed their anger to experiences of exclusion and a lack of accommodation, which further isolated them by provoking negative responses from peers.

Within hate groups, participants found a community who understood their emotions. For example, Eli shared his experience of hopelessness and disempowerment, finding solace and belonging in neo-Nazi music that resonated with the intensity of his emotions. Extreme ideologies offered a convenient explanatory framework for their feelings.

I just knew that I was angry and I needed people and I stumbled upon an online forum. . . . …I got contacted from a guy who invited me to come hang out with him, [name] and [name] and I went over and we got shit faced but I remember feeling for the first time there was a group of people who not only allowed me to feel the way I felt, but they encouraged it. —Jesse

Some participants attributed their engagement in hate-based activities to emotional resonance with the atmosphere and expression of collective anger, despite not fully aligning with the group’s ideology: “I didn’t hate Jewish people. I think I was there because the other guys were angry. I was there for the violence. I was there for the anger because I was angry.” (Morgan)

## Discussion

We examined personal and contextual factors that contribute to engagement with extreme ideologies by some autistic people. This study distinguishes itself from prior conceptual studies or single case studies by directly examining the lived experiences of autistic individuals involved in extreme ideologies. Autistic features in isolation did not explain this involvement. Childhood attachment disruptions and trauma, social marginalization, and traumatic catalytic events shaped participants’ paths toward extreme ideologies. The absence of professional recognition and support for neurodivergence, upbringing with a narrow worldview, and lack of validation further contributed to their susceptibility. Participants gravitated toward extreme ideologies to pursue their needs, interests, and abilities that were rejected or marginalized by a neurotypical society.

Our participants’ engagement in extreme ideologies can be examined through existing models of radicalization developed for broader populations, including the 3Ps^23^ model. The 3Ps model encompasses *push* factors, which relate to deprivation of a social group in terms of stigmatization and grievance; *personal* factors, including individual characteristics that confer susceptibility to radicalization; and *pull* factors, or the appeal of extremist groups and ideologies. Push factors in this context include the experience of isolation, marginalization, and a sense of injustice stemming from the societal mistreatment of autistic people. A traumatic catalytic event propelled some participants into a heightened state of hostility; such events are a common pathway to extremism.^24,25^ Importantly, our findings paint a picture of a population in distress, with systemic gaps in health and social support systems impeding access to timely diagnoses and interventions.

Personal factors included trauma and unmet needs associated with neurodivergence. Later-life mental health challenges resulting from childhood traumatic experiences have been associated with involvement in violent extremism.^26^ Autistic children face a heightened risk of potentially traumatic events due to social and communication challenges, leading to increased peer victimization and a greater likelihood of maltreatment.^27,28^ They are also more vulnerable to adversities such as parental divorce, neighborhood violence,^29^ physical abuse, and neglect,^30^ resulting in poorer mental health outcomes.^31^

Participants expressed how specific neurodivergent characteristics, such as hyperfixation, need for structure and routine, neurodivergent social style, and emotion dysregulation, contributed to unmet needs that were recognized within hate groups and propaganda. Other scholars similarly deny an overly simplistic association between autism and extremism, but concur that certain features commonly observed among autistic people can influence their participation.^13,14^ Walter et al.^14^ identified additional traits that could influence the risk of radicalization such as cognitive rigidity, difficulties identifying and understanding one’s own emotions, and struggles with abstract thinking. These features were not reported by our participants, suggesting further research incorporating broader perspectives (e.g., clinicians and family members) is needed to better understand the intersection between neurodivergence and extreme ideologies.

Many pull factors influenced engagement with hateful ideologies among our participants. Many participants had xenophobic beliefs impressed upon them by role models in their formative years, normalizing these attitudes. Findings from studies within the general population underscore that the relationship between early socialization factors and extremism is not unique to autistic individuals.^32,33^ An additional pull factor was the sense of purpose, validation, belonging offered in hate groups. Participants were motivated to engage with extreme ideologies to fulfill their unmet needs, as society failed to provide prosocial spaces, support systems, and opportunities for acceptance and purpose. A unique contribution of our work is the identification of accommodation as a pull factor. Hate groups provided a social structure that not only celebrated participants’ strengths, but also adapted to their needs, creating an appealing alternative to the exclusion and inaccessibility participants experienced in the neurotypical society.^34^

Our work touches on the complex interplay between vulnerability and agency. Many participants highlighted their vulnerability by discussing manipulation by extremist groups, possibly reflecting a desire to de-emphasize their choices to engage with hate. However, their vulnerability does not negate their agency, as vulnerability and agency are not mutually exclusive.^35^ Neurodivergence contributed to vulnerability by shaping the life experiences of participants; the resulting sense of dislocation from broader society and grievance influenced participants’ choices to adopt hateful ideologies.

There are wide-reaching implications of this work. Most importantly: autistic and otherwise neurodivergent people must feel included in society, or the appeal of transgressive groups will remain an appealing option to have these needs met. Formally identifying and supporting neurodivergent conditions can empower individuals by providing access to supports and a framework for understanding themselves.^36^ Mental health professionals should adapt early and at-risk prevention approaches to include timely and accurate identification of diagnoses as well as tailored support and accommodations. It is crucial to target those who may be seeking to exploit autistic individuals, including collaborating with online platforms and content moderators, law enforcement, and social services to monitor and counteract extremist content online and offline. Although trauma in itself is not a proven pathway to extremism for neurodivergent people, trauma and stigmatization need to be proactively identified by families, teachers, and other professionals and addressed using trauma-informed approaches that recognize the different ways that trauma can present in this population.^37^ Given the small sample size of this study, further research on pathways to radicalization, prevention, and outcomes is needed.

### Limitations

Autism is variable in terms of presentation, coping mechanisms, and perceptions.^38^ Readers should not link these participant experiences to the broad population of autistic people. Autism self-diagnoses were accepted for participation but not confirmed by diagnostic assessments. Notably, a substantial proportion of our study participants reported secondary neurodevelopmental and mental health conditions but rarely specifically discussed them. This may suggest they did not consider these conditions prominently relevant to their experiences or may reflect their assumption that researchers wished to focus on autism based on the project description. This may also be attributed to the phrasing of our questions. Our sample consisted primarily of White, American, and male participants; while this could be related to our topic, we cannot extensively comment on experiences of individuals outside these identities. Finally, participants’ candor may have been limited given the ethical duty to report any plans to harm themselves or others.

### Conclusion

Autism alone did not explain participants’ engagement with extreme ideologies; rather, the convergence of trauma, societal disenfranchisement, and the fulfillment of unmet needs influenced the experiences of our sample. Prevention and management strategies must prioritize the creation of inclusive and empowering environments, address societal mistreatment, and provide supportive structures to facilitate autistic well-being within the society.

## Authorship Confirmation Statement

M.P. conceptualized and designed the study, conducted data collection, and contributed significantly to the article draft. S.W. conducted data analysis and primarily drafted the article. The remaining authors participated in team meetings, contributed to the analysis, provided critical intellectual input, and reviewed the article. All authors have read and approved the final version of the article.

## Supplemental Material

sj-pdf-1-aua-10.1089_aut.2023.0192 — Supplemental material for Neurodivergence and the Rabbit Hole of Extremism: Uncovering Lived Experience Supplemental material, sj-pdf-1-aua-10.1089_aut.2023.0192 for Neurodivergence and the Rabbit Hole of Extremism: Uncovering Lived Experience by

## Notes

1 The “Third National Confederate battle flag” refers to the final design adopted as the official flag of the Confederate States of America during the American Civil War and holds historical significance in the context of Confederate symbolism.
