# Supplementary material for: Neurodivergence and the Rabbit Hole of Extremism: Uncovering Lived Experience
Source: Autism Adulthood. 2024 Aug 9;8(1):102–11. doi: 10.1089/aut.2023.0192 (PMC13294708; doi:10.1089/aut.2023.0192)
Supplement: sj-pdf-1-aua-10.1089_aut.2023.0192 — Supplemental material for Neurodivergence and the Rabbit Hole of Extremism: Uncovering Lived Experience [file sj-pdf-1-aua-10.1089_aut.2023.0192.pdf]

REB No.

Exploring Factors that Influence Autistic People Who Engage in Online Hate

Semi structured interview guide:

Greeting / Introduction to the study (review since was already covered in consent conversation): Thank you for joining me for this interview. We are hoping to learn from your experiences during and leading up to your involvement with (extremist disengagement support service). Remember that you don't have to answer any questions that you don't want to. If there is a question you don't understand, please ask and I will re-phrase it.

I would also like to remind you that I would have a duty to take action if you were to disclose a plan to harm yourself or someone else. In the case of self-harm, I will need to link us in to a crisis line. If you were to disclose a plan to harm someone else, I would need to notify authorities. As part of the safety plan for this study, I need to ask your current location. Please provide an address so that I can inform emergency personnel in case of emergency. After the interview is over, I will destroy this information.

Now let's begin the interview.

Q1. Tell me about your life right now?

If prompts needed: school, work, hobbies, interests. What does your day and or week usually look like?
What keeps you busy?

Q2. Tell me about people in your life who are important to you.

If prompts needed: friends, family, pets, community work
Who are the people who rely on you?
Who do you rely on?

Q3. What would you like your life to be like, in 2 years? Where do you see yourself in 5 years?

Q4. Tell me more about your family.

Who lives with you? What was it like growing up in your family?
(Feelings of being supported, does your family know who you really are? do they accept who you are?)

Q5. Please describe yourself in terms of race or heritage.

Q6. Do you identify with any sorts of in-person or online groups? For instance, cultural, heritage, or religious groups, or a group based on an interest or hobby?

Q7. What would you write in a short online biography about yourself, such as a Twitter bio?

Q8. How would you describe your relationships with people outside your immediate family?

- How often do you see them? How much time do you spend with them? What sorts of things do you do? Can you describe an example of what your time with others looks, sounds, or feels like?

Q9. How did you become involved with [extremist disengagement support service]?
(Note to interviewer: use their language for group involvement going forward)

Q10. What got you interested in this sort of thing (note their specific terminology)?

- How did you first get involved?
- Probe whether they sought it out or whether someone shared the information with them – Who? How did you know that person?
- Was there anything else was going on in your life at that time? What was going well for you? What wasn't?

Q11. Thinking back to your youth or childhood can you think of any experiences that got you interested in or made you more likely to (use their specific terminology)?

Q12. Describe your type of involvement/exposure.

- What was it like? How often did you participate in the online forums?
- Was it daily, weekly, monthly?
- How often did you post?
- How often did you comment?
- Was there a lot of commenting or dialogue around your activity?
- Were there leaders or people you admired?
- Did you become friends with anyone from online in real life?
- What other groups/sites were you engaged with online?
- What time of day did you participate? Did you engage in these things before school or work, or programs?
- Where were you when you were participating? Were other people around?

Q13. What is/was it like to participate in (reference specific group /material)?

- What sorts of topics or things do you remember reading, writing or discussing?
- What kept drawing you back to participate further?
- Did it feel like other people in the forum(s) wanted to be your friends? (reflect back description – reading / chatting / attend meetings)

Q14. Was this something you did on your own or with others (family/friends)?

Q15. Do you have friends who are interested in (reference specific group/material)?

Q16. Do you have family members who are interested in (reference specific group/material)?

Q17. We are interested in learning from people who are autistic or who have autistic traits. How do you describe yourself with regard to autism? Did you receive a diagnosis? How did you come to know that about yourself?

Q18. Do you think being (reference their self-description of autism) made you more attracted to (reference specific group/material)? How so?

Q19. Do you think that other people who are autistic thinkers might be more attracted to things like (reference specific group/material)? Why or why not?

For people who don't identify with HSF groups any longer:

Q20. What led to you get out of (reference specific group/material)? Did you make the choice yourself or did someone intervene? Was there a specific event/moment or was it a gradual process?

Q21. What advice do you have for other people who (describe factors that got this person interested initially) and who may find themselves interested or curious about (reference specific group/material)?

Q22. What advice do you have for parents / teachers / counsellors who are worried about a person becoming involved in (reference specific group/material)?
